# Metacognitive processes, situational factors, and clinical decision-making in nursing education: a quantitative longitudinal study

**DOI:** 10.1186/s12909-024-06467-y

**Published:** 2024-12-26

**Authors:** FangFang Wang, Dandan Liu, ManHong Zhang

**Affiliations:** 1 Shanxi Provincial People’s Hospital, No. 29 Shuangta East Street, Yingze District, Taiyuan City, Shanxi Province China; 2 Department of Media and Communication Studies, Faculty of Arts and Social Sciences, Universiti Malaya, Kuala Lumpur, Malaysia

**Keywords:** Nursing education, Metacognition, Clinical decision-making, Situational factors, Individual differences, Longitudinal study

## Abstract

**Objective:**

This study examined the longitudinal development of metacognitive skills and clinical decision-making abilities in nursing students, focusing on the interactions between metacognitive processes, situational factors, and individual differences.

**Methods:**

A longitudinal, quantitative design was employed, following 185 third-year nursing students from a major university in China over one academic year. Data were collected at six time points using the Metacognitive Awareness Inventory, Nursing Decision-Making Instrument, and custom-designed clinical scenario assessments. Latent Growth Curve Modeling, Multilevel Modeling, and Moderation Analysis were used to analyze the data.

**Results:**

Significant positive growth trajectories were observed for both metacognitive awareness (mean slope = 0.07, *p* < .001) and decision-making skills (mean slope = 0.08, *p* < .001). Metacognitive regulation emerged as the strongest predictor of decision-making outcomes (β = 0.188, *p* < .001 for quality; β = 0.168, *p* < .001 for efficiency). Task complexity negatively impacted decision-making quality (β = -0.129, *p* < .001), while time pressure more strongly affected efficiency (β = -0.121, *p* < .001). Cognitive style and emotional intelligence moderated the relationship between metacognitive processes and decision-making outcomes. The effectiveness of metacognitive strategies in mitigating the negative effects of situational factors varied across individuals and over time.

**Conclusion:**

This study provides robust evidence for the complex interplay between metacognitive processes, situational factors, and individual differences in the development of clinical decision-making skills among nursing students. The findings highlight the importance of tailoring educational interventions to enhance specific metacognitive skills, particularly regulation, while considering the impact of situational factors and individual cognitive styles. These insights can inform the design of more effective, personalized approaches to nursing education, potentially enhancing the preparation of nursing students for the complexities of clinical practice.

## Introduction

### Background

Clinical decision-making is a cornerstone of effective nursing practice, playing a crucial role in ensuring patient safety and quality care. In the complex and dynamic healthcare environment, nurses are continually faced with situations that require rapid assessment, critical thinking, and sound judgment [1]. The ability to make timely and accurate clinical decisions is not only essential for patient outcomes but also for the efficient functioning of healthcare systems [2].

Metacognition, often described as “thinking about thinking,” has emerged as a critical factor in enhancing decision-making skills across various domains, including nursing [3]. The concept of metacognition encompasses three primary phases: monitoring, evaluation, and regulation. Monitoring involves awareness of one’s cognitive processes during decision-making, evaluation refers to the assessment of the effectiveness of one’s thinking and decisions, and regulation involves adjusting cognitive strategies based on this monitoring and evaluation [4]. In nursing education and practice, these metacognitive processes are increasingly recognized as fundamental to developing expert clinical judgment [5].

The impact of situational factors on cognitive processes in clinical settings cannot be overstated. Factors such as task complexity, time pressure, and the availability of information significantly influence how nurses process information and make decisions [6]. These situational variables can either facilitate or hinder the application of metacognitive skills, thereby affecting the quality of clinical decision-making. Recent research has highlighted the importance of considering these contextual factors in understanding and improving nursing decision-making processes [7].

The role of individual differences in clinical decision-making has gained increasing attention in recent years. Cognitive style, defined as an individual’s preferred approach to gathering, processing, and evaluating information, represents a key factor in how nurses approach clinical situations [8]. This construct exists on a continuum from analytical (systematic, detail-oriented processing) to intuitive (holistic, pattern-based processing) approaches. Similarly, emotional intelligence—the ability to perceive, understand, and manage emotions—has emerged as a critical factor in clinical decision-making, particularly given the emotionally charged nature of healthcare environments [9]. These individual differences may interact with both metacognitive processes and situational factors to influence decision-making outcomes.

Task complexity and time pressure represent two crucial situational factors that impact clinical decision-making. Task complexity in nursing contexts encompasses multiple dimensions: the number of clinical variables to consider, the ambiguity of symptoms, the interrelatedness of patient conditions, and the potential consequences of decisions [10]. Time pressure, characterized by constraints on the duration available for assessment and decision-making, can significantly alter cognitive processing patterns and decision strategies [11]. Understanding how these situational factors interact with metacognitive processes and individual differences is essential for developing effective educational interventions.

Effective clinical decision-making is crucial for patient safety and quality care, influenced by both metacognitive processes and situational factors. Understanding the interplay between these elements is essential for developing robust nursing education programs and improving clinical practice. As healthcare becomes increasingly complex, the ability to navigate diverse clinical situations while employing advanced cognitive strategies becomes paramount for nursing professionals [12].

### Current state of research

#### Metacognition in nursing education

Metacognition, a critical component of clinical reasoning, has gained significant attention in nursing education research. The concept encompasses three primary phases: monitoring, evaluation, and regulation. Monitoring refers to the awareness of one’s cognitive processes during decision-making, enabling nurses to track their thought patterns and identify potential biases or gaps in knowledge [3]. Evaluation involves assessing the effectiveness of one’s thinking and decisions, allowing for critical reflection on the reasoning process and outcomes. Regulation, the final phase, involves adjusting cognitive strategies based on the insights gained from monitoring and evaluation, leading to improved decision-making over time [13].

The development of metacognitive skills in nursing students is a complex process that evolves throughout their educational journey. Recent studies have shown that targeted interventions can enhance metacognitive abilities, leading to improved clinical reasoning and decision-making skills [14]. For instance, reflective practice exercises, structured debriefing sessions, and the use of think-aloud protocols have been found to be effective in fostering metacognitive development [12]. Current understanding of how these metacognitive phases impact clinical reasoning suggests a strong positive correlation between metacognitive awareness and the quality of clinical decisions. Zeb et al. [15] found that nursing students with higher metacognitive skills demonstrated more sophisticated clinical reasoning patterns and were better able to navigate complex patient scenarios. However, the specific mechanisms by which each phase of metacognition influences different aspects of clinical reasoning remain an area of ongoing research.

#### Clinical decision-making in nursing

Clinical decision-making in nursing is conceptualized through various models, with the intuitive-humanist model and the information-processing model being two prominent frameworks. The intuitive-humanist model emphasizes the role of intuition and pattern recognition in decision-making, particularly in experienced nurses [1]. In contrast, the information-processing model focuses on the systematic analysis of clinical data and the application of evidence-based guidelines in reaching decisions [2]. Factors influencing decision quality and efficiency in nursing are multifaceted. Recent research has identified several key elements, including the nurse’s level of experience, the availability and quality of clinical information, the complexity of the patient’s condition, and the presence of environmental stressors [6]. Additionally, individual factors such as critical thinking skills, emotional intelligence, and cultural competence have been shown to play significant roles in shaping decision outcomes [16].

#### Individual cognitive factors in clinical decision-making

In nursing practice, cognitive style can significantly influence how practitioners gather, process, and utilize clinical information. For example, nurses with an analytical cognitive style may tend to systematically evaluate each piece of clinical data, while those with an intuitive style might rely more on pattern recognition and previous experience [17]. Cognitive style is intrinsically linked to metacognitive processes and clinical decision-making outcomes. Different nurses with different cognitive style can significantly impact their ability to monitor, evaluate, and regulate their thinking processes during clinical reasoning [18]. This relationship becomes particularly important when considering the development of clinical decision-making skills in nursing education, where students must learn to adapt their cognitive approaches while managing varying levels of cognitive demand.

#### Situational factors in clinical environments

Task complexity in nursing contexts refers to the level of difficulty and intricacy involved in patient care situations. It can be defined by factors such as the number of clinical variables to consider, the ambiguity of symptoms, and the potential for conflicting treatment priorities. Measuring task complexity in nursing has been approached through various methods, including standardized patient scenarios, complexity rating scales, and physiological measures of cognitive load [19]. Time pressure, another critical situational factor, has been shown to have significant effects on cognitive processes and decision outcomes in nursing. Studies have demonstrated that under time constraints, nurses may rely more heavily on heuristics and intuitive decision-making processes, potentially leading to errors in complex cases [20]. Conversely, moderate time pressure has been found to enhance focus and efficiency in some decision-making tasks, highlighting the nuanced nature of this factor’s impact [21]. The interplay between task complexity and time pressure creates dynamic clinical environments that challenge nurses’ cognitive resources and decision-making capabilities. Recent research has begun to explore how these situational factors interact with metacognitive processes, suggesting that effective metacognition may serve as a buffer against the negative impacts of high complexity and time pressure [12].

### Research gaps

Despite significant advancements in understanding metacognition, clinical decision-making, and situational factors in nursing, several critical research gaps persist. These gaps limit our comprehensive understanding of how these elements interact and evolve over time, particularly in the context of nursing education.

While individual components of metacognition, situational factors, and decision-making have been studied extensively, there is a notable lack of research examining their complex interactions. Specifically, the interplay between different phases of metacognition (monitoring, evaluation, and regulation) and various situational factors (such as task complexity and time pressure) in clinical decision-making processes remains poorly understood [3]. For instance, it is unclear how metacognitive monitoring might be affected by high task complexity, or how time pressure influences metacognitive regulation strategies. Furthermore, the dynamic nature of these interactions over the course of a nurse’s education and early career has not been adequately explored. Zeb et al. [15] highlight the need for research that captures how the relationship between metacognitive processes and decision-making outcomes may change as nursing students gain more clinical experience and encounter increasingly complex patient scenarios.

The majority of existing research in this field relies on cross-sectional designs or short-term interventions. There is a significant lack of longitudinal studies that track the development of metacognitive skills, decision-making abilities, and their interactions with situational factors over extended periods in nursing education [14]. This gap is particularly problematic because the development of clinical reasoning skills is a gradual process that unfolds over years of education and practice. Longitudinal studies are crucial for understanding how metacognitive strategies evolve in response to repeated exposure to various clinical situations and how this evolution impacts decision-making quality over time. Such research could provide valuable insights into the optimal timing and methods for metacognitive skill development interventions throughout nursing education programs [12].

While metacognition is often studied as a unified construct, there is limited research on how its specific phases (monitoring, evaluation, and regulation) interact individually with situational factors and influence decision-making in nursing contexts. Lopez-Morinigo et al. [4] note that understanding these phase-specific interactions is crucial for developing targeted interventions to enhance clinical reasoning skills. For example, it remains unclear whether certain phases of metacognition are more susceptible to the effects of time pressure or task complexity. Additionally, there is a lack of research examining how the effectiveness of different metacognitive phases in decision-making might vary across different types of clinical scenarios or at different stages of a nurse’s education and career.

While the importance of situational factors in clinical decision-making is well-recognized, research often fails to adequately integrate these factors when studying metacognitive processes in nursing. Oh et al. [7] argue that this oversight limits our understanding of how metacognitive skills are applied in real-world clinical environments, where situational factors play a significant role. There is a need for studies that systematically manipulate situational factors such as task complexity and time pressure while assessing metacognitive processes and decision-making outcomes. Such research could provide insights into how nurses adapt their metacognitive strategies in response to varying situational demands and how these adaptations impact the quality of clinical decisions.

The theoretical basis for examining these relationships stems from several key frameworks. The Information Processing Theory [22] suggests that cognitive processes are influenced by both individual characteristics and environmental factors. Similarly, the Cognitive Continuum Theory [23] proposes that decision-making modes vary along an intuitive-analytical continuum depending on task characteristics and individual preferences. These theoretical frameworks, combined with empirical evidence from nursing education research, support the investigation of how metacognitive processes interact with individual differences and situational factors in clinical decision-making.

Addressing these research gaps is crucial for enhancing our understanding of how to improve clinical decision-making skills in nursing education. By exploring the complex interactions between metacognition phases, situational factors, and decision-making processes over time, researchers can provide valuable insights for developing more effective educational interventions. This knowledge could inform the design of nursing curricula that better prepare students for the cognitive demands of clinical practice, ultimately leading to improved patient care and safety. Furthermore, a more nuanced understanding of these interactions could guide the development of targeted support strategies for nursing students and early career nurses, helping them navigate the challenges of clinical decision-making in complex and dynamic healthcare environments. As healthcare continues to evolve and become more complex, addressing these research gaps becomes increasingly important for advancing nursing education and practice.

### Research aim and questions

The purpose of this study is to examine the dynamic relationships between the phases of metacognition, situational factors, and clinical decision-making abilities in nursing students over an extended period. By tracking these relationships throughout the course of nursing education, this research aims to shed light on how metacognitive skills develop and interact with decision-making processes in various clinical contexts. This longitudinal approach allowed for a nuanced understanding of how these critical cognitive abilities evolve as students gain experience and encounter increasingly complex clinical scenarios.

To address the identified gaps and fulfill the study’s purpose, the following research questions have been formulated:

1. How do the different phases of metacognition (monitoring, evaluation, regulation) influence the quality and efficiency of clinical decision-making in nursing students over time?
2. How do situational factors (task complexity, time pressure) affect the relationship between each phase of metacognition and clinical decision-making?
3. How do individual differences in cognitive style and emotional intelligence, influence the development of metacognitive abilities and their impact on clinical decision-making skills?
4. How do the three phases of metacognitive abilities and their respective impacts on clinical decision-making evolve as nursing students gain more experience?

## Methodology

### Research design

This study employs a quantitative longitudinal design to investigate the development of metacognitive phases and decision-making skills in nursing students over time. The longitudinal approach is chosen for its unique ability to track developmental trajectories and capture changes in cognitive processes throughout a crucial period of nursing education [24]. The study followed participants over one academic year, corresponding to the third year of their nursing education. This timeframe is selected to capture a critical period during which nursing students are exposed to more advanced clinical reasoning and practical experiences. Data collection occurred at six time points throughout the academic year: three times per semester, at the beginning, middle, and end of each semester.

A longitudinal design offers several advantages for this study. Firstly, it allows for the assessment of intra-individual changes over time, providing insights into how each student’s metacognitive abilities and decision-making skills develop within a concentrated period. Secondly, it enables the examination of inter-individual differences in these developmental trajectories, which is crucial for understanding the role of individual factors in cognitive skill acquisition. Finally, this design permits the investigation of time-varying covariates, such as situational factors, and their impact on the relationships between metacognition and clinical decision-making [25].

### Participants

The study recruited 200 nursing students from Shanxi Medical University, Shanxi province, China. A power analysis was conducted using G*Power software [26], assuming a medium effect size (*f* = 0.25), an alpha level of 0.05, and a desired power of 0.80 for repeated measures ANOVA with six measurement points. The analysis indicated that a minimum sample size of 166 would be required. The target sample of 200 accounts for potential attrition over the one-year study period, with an estimated retention rate of 90% based on similar short-term longitudinal studies in nursing education [27]. Inclusion criteria specify that participants must be third-year nursing students at Shanxi Medical University at the commencement of the study. Exclusion criteria include students with prior nursing experience or those following non-traditional educational pathways, as these factors could introduce confounding variables that may affect the developmental trajectories under investigation. Recruitment procedures involved close collaboration with the nursing school administration at Shanxi Medical University. Information sessions were conducted to introduce the study to potential participants, explaining its purpose, procedures, and the commitment required over the academic year. These sessions also addressed any questions or concerns students may have about participation. Following the information sessions, interested students were provided with detailed consent forms outlining the study’s objectives, potential risks and benefits, confidentiality measures, and the voluntary nature of participation.

### Measures and instruments

This study employed a comprehensive set of validated instruments to measure metacognitive processes, decision-making abilities, and related cognitive constructs. Additionally, it incorporates custom-designed simulation scenarios to manipulate task complexity and time pressure.

The Metacognitive Awareness Inventory (MAI), developed by [28] and validated for use in nursing education by [29], were used to assess participants’ metacognitive awareness. The MAI provides subscores for the three key phases of metacognition: monitoring, evaluation, and regulation. This 52-item self-report questionnaire has demonstrated high internal consistency (Cronbach’s α = 0.91) and test–retest reliability (*r* = 0.88) in recent studies with nursing students [29]. The Chinese version of the MAI, validated by [30], were used in this study, showing comparable psychometric properties (Cronbach’s α = 0.89). Monitoring is operationalized as the real-time awareness and tracking of cognitive processes during clinical decision-making, measured through items such as “I ask myself periodically if I am meeting my goals.” Evaluation encompasses the assessment and judgment of cognitive strategies and decision outcomes, while regulation refers to the adjustment and control of cognitive strategies based on monitoring and evaluation outcomes. Factor analysis has confirmed the three-dimensional structure of the instrument (CFI = 0.92, RMSEA = 0.058), with factor loadings ranging from 0.45 to 0.81 across all dimensions.

To capture specific metacognitive activities in clinical scenarios, the Metacognitive Activities Inventory (MCA-I) were administered. Developed by [31] for use in healthcare education, the MCA-I has shown strong construct validity (CFI = 0.95, RMSEA = 0.06) and internal consistency (Cronbach’s α = 0.88) in previous studies. The MCA-I provides a more context-specific measure of metacognitive processes during clinical decision-making tasks, complementing the broader assessment provided by the MAI.

The quality and efficiency of clinical decisions were measured using the Nursing Decision-Making Instrument (NDMI), originally developed by [32] and recently updated by [33]. The updated NDMI has demonstrated good internal consistency (Cronbach’s α = 0.85) and construct validity (CFI = 0.93, RMSEA = 0.05) in its validation studies. The Chinese version of the NDMI, adapted and validated by Liu et al. (2022), were used in this study (Cronbach’s α = 0.83). Decision quality is operationalized through three components: information gathering, option evaluation, and decision appropriateness, each measured through validated subscales [34]. Decision efficiency encompasses time management, resource utilization, and decision speed dimensions. The instrument’s factor structure has been confirmed through rigorous confirmatory factor analysis, with all items showing significant loadings (> 0.40) on their respective factors.

The Emotional Intelligence Scale (EIS) developed by [35] were used to measure participants’ ability to perceive, use, understand, and manage emotions. This scale has demonstrated good internal consistency (Cronbach’s α = 0.89) and predictive validity in nursing contexts [36]. The Chinese version of the EIS, validated by [37], were used in this study (Cronbach’s α = 0.87). The instrument measures four distinct dimensions: self-emotion appraisal, others’ emotion appraisal, use of emotion, and regulation of emotion. Each dimension has demonstrated strong psychometric properties, with factor loadings ranging from 0.58 to 0.84 and significant convergent validity with related constructs.

To assess individual preferences in information processing, the Cognitive Style Index (CSI) developed by [38] and recently updated by [39] were employed. The updated CSI has shown strong psychometric properties, including high internal consistency (Cronbach’s α = 0.84) and test–retest reliability (*r* = 0.90). The Chinese version of the CSI, adapted and validated by [40], were used in this study (Cronbach’s α = 0.82). The instrument measures both analytical and intuitive cognitive styles, with demonstrated construct validity through confirmatory factor analysis (CFI = 0.91, RMSEA = 0.06).

A crucial component of this study is the Task Complexity and Time Pressure Manipulations in Simulations. These custom-designed scenarios were developed based on the framework proposed by [19] for measuring task complexity in nursing simulations. The scenarios were vary systematically in complexity (low vs. high) and time pressure (low vs. high), resulting in four distinct types of simulations. Key aspects of these manipulations include:

1. Complexity factors: number of relevant cues, interrelatedness of cues, clarity and stability of the patient’s condition, and number of treatment options.
2. Time pressure factors: time allowed for initial assessment, frequency of patient condition updates, time allowed for treatment decisions, and presence of concurrent tasks or interruptions.

Four standardized clinical scenarios were developed to systematically assess clinical decision-making under varying conditions of complexity and time pressure. The scenarios represented common medical-surgical nursing situations: post-operative care, respiratory distress, diabetic emergency, and altered mental status. These core scenarios were selected based on their prevalence in clinical practice and their amenability to complexity manipulation.

Task complexity was operationalized through two primary dimensions: information load and information diversity. Information load was manipulated through the number of clinical variables (ranging from 4 to 12 elements), while information diversity was varied through the presence of comorbidities and medication interactions. High-complexity scenarios incorporated multiple interacting symptoms, complicated medication histories, and concurrent care requirements. In contrast, low-complexity scenarios presented clear, single-system issues with linear symptom presentations and straightforward treatment pathways.

Time pressure was systematically varied through two mechanisms: assessment windows (15 versus 5 min) and the frequency of patient condition changes. High time-pressure conditions featured shorter assessment windows and more frequent updates to patient status, while low time-pressure conditions allowed more time for assessment and featured stable patient presentations.

The scenarios underwent rigorous validation through a two-stage process. Initially, an expert panel (*n* = 5) evaluated each scenario using a standardized complexity rating tool, achieving high inter-rater reliability (ICC = 0.87). Subsequently, the scenarios were pilot-tested with senior nursing students (*n* = 20) who were not participating in the main study. Implementation utilized high-fidelity simulation with standardized patients, incorporating dynamic vital sign changes, laboratory results, and clinical documentation requirements calibrated to each scenario’s complexity level.

### Data collection procedures

**Fig. 1:**
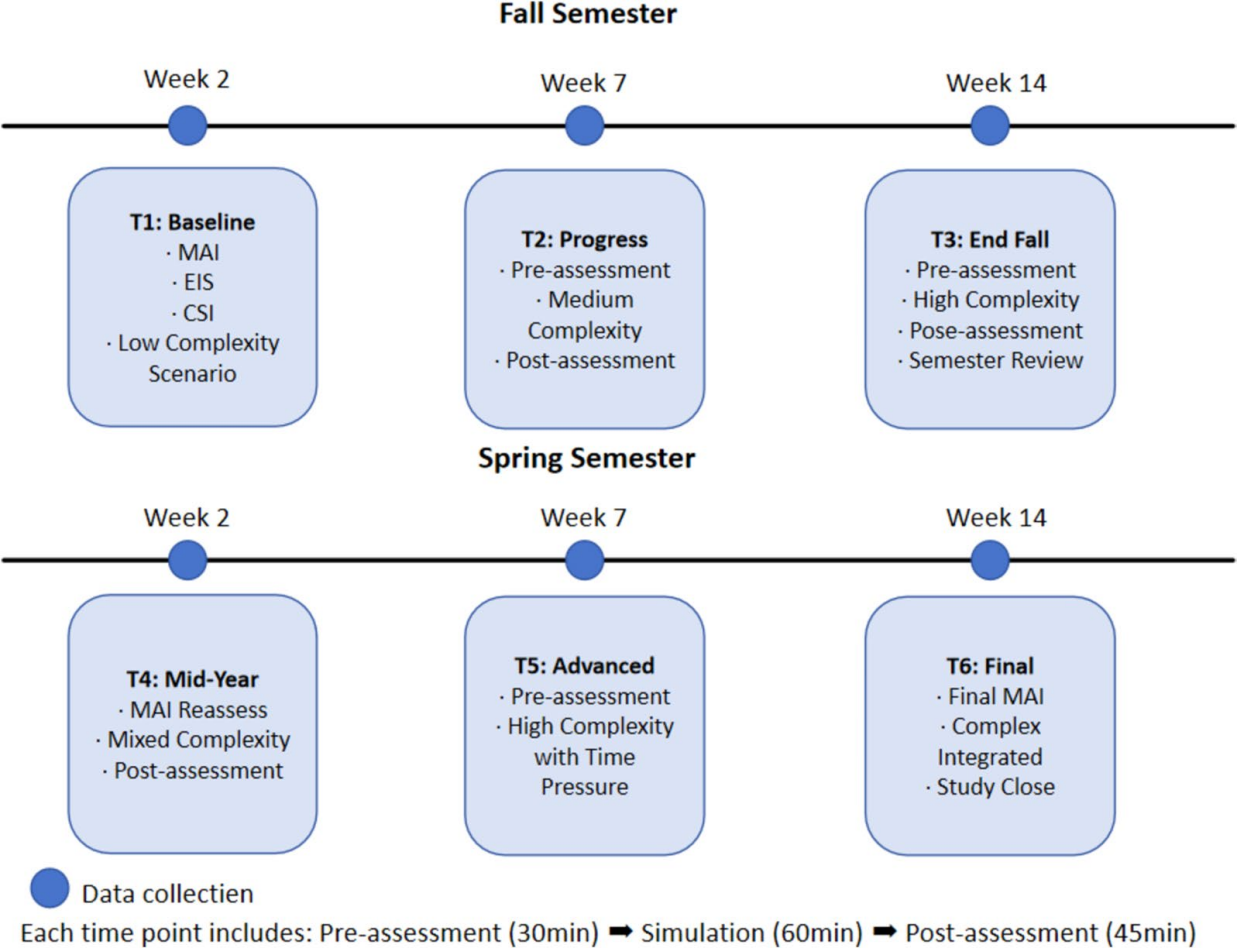
Data collection timeline spanning two semesters

At each data collection point, participants were engaged in simulation scenarios designed to manipulate task complexity and time pressure. These scenarios, developed based on the framework proposed by [41], were presented four distinct conditions: (1) Low Complexity/Low Time Pressure, (2) Low Complexity/High Time Pressure, (3) High Complexity/Low Time Pressure, and (4) High Complexity/High Time Pressure. This design allowed for the systematic examination of how situational factors influence metacognitive processes and decision-making outcomes. Pre-simulation assessments included the Metacognitive Awareness Inventory (MAI [29];), Emotional Intelligence Scale (EIS; [37]), and Cognitive Style Index (CSI; [42]). These measures provided insights into participants’ baseline metacognitive awareness, emotional intelligence, and cognitive processing preferences. Additionally, trained observers used standardized observation protocols to record participants’ behaviors and decision-making processes. These observational measures provided objective data to complement self-reported measures. Post-simulation assessments included the Nursing Decision-Making Instrument (NDMI; [43]) and the Metacognitive Activities Inventory (MCA-I; [31]). These instruments captured participants’ decision-making quality and efficiency, as well as their engagement in specific metacognitive activities during the clinical scenarios. As illustrated in Fig. 1, the data collection process spanned one academic year with six time points (three per semester at Weeks 2, 7, and 14), each incorporating a standardized sequence of pre-assessment, simulation, and post-assessment activities. At each time point, participants completed specific assessment batteries and clinical scenarios of increasing complexity, progressing from baseline measurements with low-complexity scenarios (T1) to final integrated assessments with complex scenarios (T6), ensuring systematic capture of developmental trajectories in metacognitive and decision-making abilities.

Of the initial 200 participants, 185 (92.5%) completed all six time points of the study. This final sample was used for all subsequent analyses. The high retention rate (92.5%) across six time points was achieved through careful integration of data collection with the regular academic curriculum. All assessments were conducted during scheduled “Clinical Decision-Making Development” sessions, which were part of the mandatory third-year nursing curriculum. These sessions occurred in classroom settings equipped with simulation facilities at six predetermined points throughout the academic year: weeks 2, 7, and 14 of both fall and spring semesters. To maintain objectivity and minimize potential bias, course coordinators were involved only in study planning, while data collection was conducted exclusively by four trained research assistants who had no teaching or assessment responsibilities with the participants. Additional retention strategies included offering make-up sessions within the same week for absent students and maintaining consistent research assistants throughout the study to build rapport. Of the initial 200 participants, only 15 did not complete all six time points due to medical leave (*n* = 7), academic leave (*n* = 4), or voluntary withdrawal (*n* = 4).

### Data analysis

All statistical analyses were conducted using Mplus version 8.6 for Latent Growth Curve Modeling (LGCM) and R version 4.2.1 with the lme4 package (version 1.1–31) for multilevel modeling [44]. Power analyses were conducted separately for each analytical approach to ensure adequate sample size. For LGCM, Monte Carlo simulation [45] in Mplus indicated that a sample size of 160 would achieve power of 0.80 to detect significant growth parameters with α = 0.05, assuming six time points and expected attrition of 15%. This estimation was based on previous longitudinal studies in nursing education showing medium effect sizes (δ = 0.40–0.50) for metacognitive development.

For multilevel modeling, simulation-based power analysis using the simr package in R demonstrated that a minimum sample size of 155 participants with six repeated measurements (resulting in 930 observations) would provide adequate power (0.80) to detect small to medium effects (δ = 0.35) in cross-level interactions, assuming an intraclass correlation coefficient of 0.20 based on pilot data. Our final sample of 185 participants (1,110 observations) exceeded both minimum requirements, with a retention rate of 92.5% supporting the robustness of our analyses. The nested structure of our data required a two-level analysis framework. Level 1 (within-person level) included time-varying variables measured at each assessment point: Time (coded as 0–5 for the six measurement occasions), Metacognitive processes (monitoring, evaluation, regulation scores), Situational factors (task complexity, time pressure) and Decision-making outcomes (quality and efficiency). Level 2 (between-person level) included stable individual characteristics measured at baseline: Cognitive style (analytical vs. intuitive), Emotional intelligence Demographics (age, prior experience) and Baseline metacognitive ability.

LGCM was employed to analyze developmental trajectories, with separate models for metacognitive abilities and decision-making skills. Model fit was evaluated using standard criteria: CFI > 0.95, TLI > 0.95, RMSEA < 0.06, and SRMR < 0.08 [46]. Multilevel modeling utilized Restricted Maximum Likelihood estimation, following a sequential approach from null model to full model with predictors and interactions. Model comparisons employed likelihood ratio tests and AIC/BIC criteria, with all continuous predictors grand-mean centered for interpretation clarity. All analyses followed established guidelines for longitudinal data analysis in educational research.

## Results

### Descriptive statistics

The final sample consisted of 185 third-year nursing students (85.9% female) with a mean age of 20.8 years (SD = 1.3). Only 5.9% of participants reported having prior clinical experience. The participant characteristics is reported in Table 1.

**Table 1:** Participant characteristics

Characteristic	n (%) or M (SD)
Age (years)	20.8 (1.3)
Gender	
Female	159 (85.9%)
Male	26 (14.1%)
Prior clinical experience	
Yes	11 (5.9%)
No	174 (94.1%)

Table 2 presents descriptive statistics and correlations among key variables at the final time point (T6). The mean scores for metacognitive abilities (monitoring, evaluation, and regulation) ranged from 3.68 to 3.82 on a 5-point scale, indicating moderately high levels of metacognitive awareness among participants. Decision-making quality (M = 3.71, SD = 0.62) showed a slightly higher mean score compared to decision-making efficiency (M = 3.53, SD = 0.65), suggesting that participants perceived themselves as making somewhat better-quality decisions than efficient ones.

**Table 2:** Descriptive statistics and correlations of key variables at final time point (T6)

Variable	M (SD)	1	2	3	4	5	6
MAI Monitoring	3.82 (0.67)	-					
MAI Evaluation	3.75 (0.71)	.63**	-				
MAI Regulation	3.68 (0.73)	.59**	.61**	-			
NDMI Quality	3.71 (0.62)	.42**	.38**	.45**	-		
NDMI Efficiency	3.53 (0.65)	.35**	.31**	.39**	.68**	-	
EIS	3.89 (0.58)	.29**	.27**	.33**	.25**	.22**	-

*N* = 185

*MAI *Metacognitive Awareness Inventory*, NDMI *Nursing Decision-Making Instrument*, EIS *Emotional Intelligence Scale

**p < .01

All metacognitive phases (monitoring, evaluation, and regulation) were positively correlated with decision-making quality and efficiency, with correlations ranging from weak to moderate (rs = 0.31 to 0.45, *ps* < 0.01). Among the metacognitive phases, regulation showed the strongest correlations with both decision-making quality (*r *= 0.45, *p* < 0.01) and efficiency (*r* = 0.39, *p* < 0.01). This pattern suggests that the ability to regulate one’s cognitive processes may be particularly important for effective clinical decision-making. Emotional intelligence showed weak to moderate positive correlations with both metacognitive phases (rs = 0.27 to 0.33, *ps* < 0.01) and decision-making outcomes (rs = 0.22 to 0.25, *ps* < 0.01). These correlations, while significant, were lower than those between metacognitive phases and decision-making outcomes, indicating that emotional intelligence may play a supportive but less central role in clinical decision-making compared to metacognitive abilities. The intercorrelations among metacognitive phases were moderate to strong (rs = 0.59 to 0.63, *ps* < 0.01), suggesting that while these phases are related, they also represent distinct aspects of metacognition. Similarly, decision-making quality and efficiency showed a strong correlation (*r* = 0.68, *p* < 0.01), indicating that these two aspects of decision-making are closely related but not redundant.

### Developmental trajectories

**Fig. 2:**
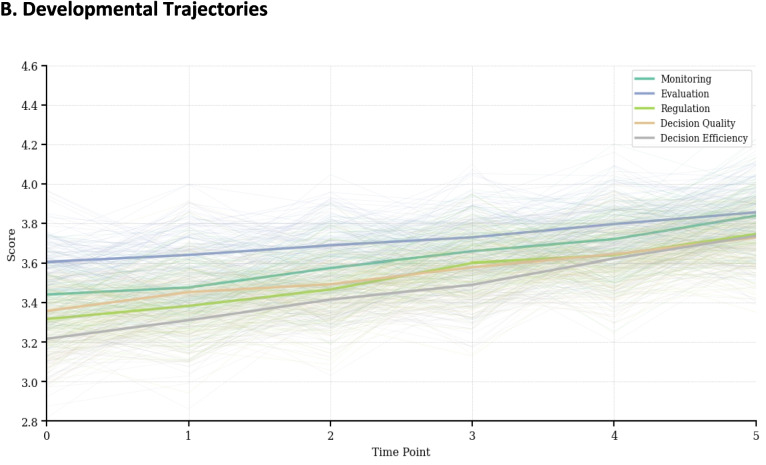
Developmental trajectories of metacognitive abilities and decision-making skills

The Latent Growth Curve Models (LGCM) for all variables demonstrated good fit to the data, with CFI and TLI values above 0.98, RMSEA values below 0.06, and SRMR values below 0.04. These fit indices suggest that the linear growth models adequately represent the developmental trajectories of metacognitive abilities and decision-making skills over the course of the study. Examination of the growth parameters revealed significant positive growth rates (slopes) for all variables, indicating that nursing students’ metacognitive abilities and decision-making skills improved over time. The growth rates varied considerably across variables, ranging from 0.05 to 0.10 points per time point. Decision-making efficiency showed the steepest growth (0.10), followed by regulation (0.09), suggesting that students’ ability to make quick decisions and regulate their cognitive processes improved more rapidly than other skills (Fig. 2).

Among the metacognitive abilities, regulation demonstrated the highest growth rate (0.09), followed by monitoring (0.08) and evaluation (0.05). This pattern suggests that students’ ability to regulate and monitor their cognitive processes improved more rapidly than their evaluation skills. The substantial difference in growth rates among metacognitive abilities indicates that these skills may develop at different paces during nursing education. Initial levels (intercepts) varied notably across variables, with evaluation showing the highest initial level (3.58) and decision efficiency the lowest (3.21). This pattern suggests that at the beginning of the study, students perceived themselves as more capable in metacognitive evaluation but less proficient in making efficient clinical decisions. All variables demonstrated significant variance in both intercepts and slopes, indicating substantial individual differences in both starting points and rates of change. The variance in slopes was particularly pronounced for regulation and decision efficiency, suggesting greater individual variability in the development of these skills (Tables 3 and 4).

**Table 3:** Latent growth curve model results for metacognitive abilities and decision-making skills

Variable	Intercept	Slope	Intercept Variance	Slope Variance	I-S Covariance
Monitoring	3.42***	0.08***	0.41***	0.01***	−0.02**
	(0.07)	(0.01)	(0.06)	(0.002)	(0.006)
Evaluation	3.58***	0.05***	0.38***	0.01***	−0.01*
	(0.06)	(0.01)	(0.05)	(0.002)	(0.005)
Regulation	3.29***	0.09***	0.47***	0.02***	−0.03**
	(0.07)	(0.01)	(0.07)	(0.003)	(0.008)
Decision Quality	3.37***	0.07***	0.35***	0.01***	−0.02**
	(0.06)	(0.01)	(0.05)	(0.002)	(0.006)
Decision Efficiency	3.21***	0.10***	0.39***	0.02***	−0.03**
	(0.07)	(0.01)	(0.06)	(0.003)	(0.007)

*p < .05, **p < .01, ***p < .001

**Table 4:** Model fit statistics

Variable	χ^2^ (df)	CFI	TLI	RMSEA	SRMR
Monitoring	18.72 (12)	0.989	0.987	0.052	0.034
Evaluation	22.45 (12)	0.985	0.982	0.058	0.038
Regulation	20.13 (12)	0.987	0.984	0.055	0.036
Decision Quality	16.89 (12)	0.991	0.990	0.048	0.032
Decision Efficiency	19.76 (12)	0.988	0.986	0.054	0.035

Standard errors in parentheses

*I-S Covariance* Intercept-Slope Covariance, *CFI* Comparative Fit Index, *TLI* Tucker-Lewis Index, *RMSEA* Root Mean Square Error of Approximation, *SRMR* Standardized Root Mean Square Residual

The negative intercept-slope covariances observed for all variables suggest that students with higher initial levels tended to show slower growth rates. However, the magnitude of these covariances varied across variables, with regulation and decision efficiency showing stronger negative relationships between initial levels and growth rates compared to other variables.

### Metacognitive processes and decision-making quality and efficiency

The multilevel analyses revealed distinct patterns in the development and influences on decision-making quality and efficiency. Intraclass correlation coefficients were similar for both outcomes (ICCquality = 0.612, ICCefficiency = 0.610), indicating that approximately 61% of variance was attributable to between-person differences (Tables 5 and 6).

**Table 5:** Multilevel models predicting decision-making quality

Fixed Effects	Model 1	Model 2	Model 3
Level 1 (Within-Person)			
Intercept	3.412***	3.376***	3.401***
	(0.048)	(0.046)	(0.045)
Time	0.076***	0.069***	0.065***
	(0.011)	(0.010)	(0.010)
Monitoring	0.183***	0.172***	0.165***
	(0.035)	(0.033)	(0.032)
Evaluation	0.156***	0.147***	0.141***
	(0.033)	(0.031)	(0.030)
Regulation	0.208***	0.195***	0.188***
	(0.036)	(0.034)	(0.033)
Task Complexity		−0.135***	−0.129***
		(0.029)	(0.028)
Time Pressure		−0.108***	−0.103***
		(0.028)	(0.027)
Level 2 (Between-Person)			
Cognitive Style	−0.087**	−0.082**	−0.079**
	(0.029)	(0.028)	(0.027)
Emotional Intelligence	0.104***	0.098***	0.095***
	(0.027)	(0.027)	(0.026)
Prior Experience	0.076*	0.072*	0.069*
	(0.031)	(0.030)	(0.029)
Cross-Level Interactions			
Monitoring × Task Complexity			0.057*
			(0.024)
Regulation × Task Complexity			0.068**
			(0.025)
Random Effects			
σ^2^ (Level 1 variance)	0.198	0.182	0.175
τ00 (Level 2 variance)	0.312	0.285	0.276
ICC	0.612	0.610	0.612
Model Fit			
−2 Log Likelihood	2178.4	2131.7	2116.5
AIC	2198.4	2157.7	2148.5
BIC	2223.1	2187.4	2189.2
Marginal R^2^	0.287	0.324	0.341
Conditional R^2^	0.658	0.682	0.694

Standard errors in parentheses. All continuous predictors are grand-mean centered

Level 1 *N* = 1,110 observations (185 participants × 6 time points)

Level 2 *N* = 185 participants

*p < .05, **p < .01, ***p < .001

**Table 6:** Multilevel models predicting decision-making efficiency

Fixed Effects	Model 1	Model 2	Model 3
Level 1 (Within-Person)			
Intercept	3.235***	3.201***	3.219***
	(0.051)	(0.048)	(0.047)
Time	0.092***	0.085***	0.081***
	(0.012)	(0.011)	(0.011)
Monitoring	0.162***	0.153***	0.147***
	(0.037)	(0.034)	(0.033)
Evaluation	0.139***	0.131***	0.126***
	(0.035)	(0.032)	(0.031)
Regulation	0.185***	0.174***	0.168***
	(0.038)	(0.035)	(0.034)
Task Complexity		−0.118***	−0.113***
		(0.030)	(0.029)
Time Pressure		−0.127***	−0.121***
		(0.029)	(0.028)
Level 2 (Between-Person)			
Cognitive Style	−0.076**	−0.072**	−0.069**
	(0.030)	(0.029)	(0.028)
Emotional Intelligence	0.093***	0.088***	0.084***
	(0.028)	(0.027)	(0.026)
Prior Experience	0.082*	0.078*	0.075*
	(0.032)	(0.031)	(0.030)
Cross-Level Interactions			
Monitoring × Time Pressure			0.052*
			(0.025)
Regulation × Time Pressure			0.061*
			(0.026)
Random Effects			
σ^2^ (Level 1 variance)	0.215	0.196	0.189
τ00 (Level 2 variance)	0.337	0.306	0.297
ICC	0.610	0.609	0.611
Model Fit			
−2 Log Likelihood	2231.7	2179.5	2165.2
AIC	2251.7	2205.5	2197.2
BIC	2276.4	2235.2	2237.9
Marginal R^2^	0.264	0.307	0.323
Conditional R^2^	0.642	0.673	0.684

Standard errors in parentheses. All continuous predictors are grand-mean centered

Level 1 *N* = 1,110 observations (185 participants × 6 time points)

Level 2 *N* = 185 participants

*p < .05, **p < .01, ***p < .001

Metacognitive processes showed consistent positive effects across both outcomes, with regulation emerging as the strongest predictor for both quality (β = 0.188, *p* < 0.001) and efficiency (β = 0.168, *p* < 0.001), followed by monitoring (βquality = 0.165, βefficiency = 0.147, *p* < 0.001) and evaluation (βquality = 0.141, βefficiency = 0.126, *p* < 0.001). Notably, the effects were consistently stronger for quality than efficiency, suggesting metacognitive processes may have greater influence on decision accuracy than speed.

Situational factors demonstrated differential effects: task complexity had a stronger negative impact on quality (β = −0.129, *p* < 0.001), while time pressure more strongly affected efficiency (β = −0.121, *p* < 0.001). This pattern suggests that complex situations primarily compromise decision accuracy, whereas time constraints mainly impact decision speed. Temporal improvement was more pronounced for efficiency (β = 0.081, *p* < 0.001) than quality (β = 0.065, *p* < 0.001), indicating faster development of speed-related skills.

Individual differences showed consistent patterns across both outcomes but with varying magnitudes. Cognitive style demonstrated negative effects (βquality = −0.079, βefficiency = −0.069, *p* < 0.01), suggesting intuitive thinkers generally performed lower on both outcomes. Emotional intelligence showed positive effects (βquality = 0.095, βefficiency = 0.084, *p* < 0.001), with stronger influence on quality than efficiency. Prior experience showed slightly stronger effects on efficiency (β = 0.075, *p* < 0.05) than quality (β = 0.069, *p* < 0.05).

The pattern of interactions differed between outcomes: for quality, regulation × task complexity showed the strongest effect (β = 0.068, *p* < 0.01), while for efficiency, regulation × time pressure was most influential (β = 0.061, *p* < 0.05). These patterns suggest that metacognitive regulation helps maintain performance quality under complexity and supports efficiency under time pressure. The final models explained similar proportions of variance, with slightly better prediction for quality (Marginal *R*^2^ = 0.341, Conditional *R*^2^ = 0.694) than efficiency (Marginal *R*^2^ = 0.323, Conditional *R*^2^ = 0.684). This suggests that while our measured variables captured key influences on both outcomes, additional factors might particularly affect decision-making efficiency.

### Moderation effects

The analysis of moderation effects revealed distinct patterns for cognitive style and emotional intelligence in their influence on decision-making processes. For cognitive style (Table 7), the ICC of 0.613 indicated that 61.3% of the variance was attributable to between-person differences. At Level 2, cognitive style showed a consistent negative main effect (β = −0.079, *p* < 0.01) on decision-making quality. More importantly, the cross-level interactions revealed that cognitive style significantly moderated the effects of both monitoring (β = 0.047, *p* < 0.05) and regulation (β = 0.055, *p* < 0.01) on decision-making quality, while its interaction with evaluation was not significant (β = 0.036, *p* > 0.05).

**Table 7:** Moderation effects of cognitive style on decision-making quality

Fixed Effects	Model 1	Model 2	Model 3
Level 1 (Within-Person)			
Intercept	3.387***	3.402***	3.415***
	(0.047)	(0.046)	(0.045)
Monitoring	0.168***	0.162***	0.157***
	(0.034)	(0.033)	(0.032)
Evaluation	0.143***	0.138***	0.134***
	(0.032)	(0.031)	(0.030)
Regulation	0.192***	0.185***	0.179***
	(0.035)	(0.034)	(0.033)
Level 2 (Between-Person)			
Cognitive Style	−0.087**	−0.082**	−0.079**
	(0.029)	(0.028)	(0.027)
Emotional Intelligence		0.104***	0.098***
		(0.027)	(0.026)
Cross-Level Interactions			
Monitoring × Cognitive Style	0.053*	0.049*	0.047*
	(0.023)	(0.022)	(0.021)
Evaluation × Cognitive Style	0.041	0.038	0.036
	(0.022)	(0.021)	(0.020)
Regulation × Cognitive Style	0.062**	0.058**	0.055**
	(0.024)	(0.023)	(0.022)
Random Effects			
σ^2^ (Level 1 variance)	0.183	0.176	0.170
τ00 (Level 2 variance)	0.289	0.278	0.269
ICC	0.612	0.612	0.613
Model Fit			
−2 Log Likelihood	2138.6	2121.3	2103.7
AIC	2164.6	2151.3	2137.7
BIC	2205.3	2205.0	2204.4
Marginal R^2^	0.319	0.337	0.355
Conditional R^2^	0.677	0.689	0.701

*p < .05, **p < .01, ***p < .001

For emotional intelligence (Table 8), the analysis demonstrated a slightly lower ICC of 0.608, with significant effects at both levels. At the between-person level, emotional intelligence showed a positive main effect (β = 0.084, *p* < 0.001) on decision-making efficiency. The cross-level interactions revealed that emotional intelligence significantly moderated the relationships between metacognitive processes and decision-making efficiency, with stronger effects for regulation (β = 0.041, *p* < 0.05) and monitoring (β = 0.038, *p* < 0.05) than for evaluation (β = 0.027, *p* > 0.05). The final models demonstrated good fit, with Model 3 explaining 35.5% of the variance in decision-making quality (Table 7, Marginal *R*^2^ = 0.355) and 33.2% of the variance in decision-making efficiency (Table 8, Marginal *R*^2^ = 0.332). The inclusion of random effects substantially improved the explained variance in both models (Conditional *R*^2^ = 0.701 and 0.686, respectively).

**Table 8:** Moderation effects of emotional intelligence on decision-making efficiency

Fixed Effects	Model 1	Model 2	Model 3
Level 1 (Within-Person)			
Intercept	3.208***	3.223***	3.239***
	(0.049)	(0.048)	(0.047)
Monitoring	0.149***	0.144***	0.139***
	(0.035)	(0.034)	(0.033)
Evaluation	0.127***	0.123***	0.119***
	(0.033)	(0.032)	(0.031)
Regulation	0.171***	0.165***	0.159***
	(0.036)	(0.035)	(0.034)
Level 2 (Between-Person)			
Cognitive Style	−0.076**	−0.072**	−0.069**
	(0.030)	(0.029)	(0.028)
Emotional Intelligence	0.093***	0.088***	0.084***
	(0.028)	(0.027)	(0.026)
Cross-Level Interactions			
Monitoring × Emotional Intelligence	0.042*	0.040*	0.038*
	(0.022)	(0.021)	(0.020)
Evaluation × Emotional Intelligence	0.031	0.029	0.027
	(0.021)	(0.020)	(0.019)
Regulation × Emotional Intelligence	0.046*	0.043*	0.041*
	(0.023)	(0.022)	(0.021)
Random Effects			
σ^2^ (Level 1 variance)	0.199	0.192	0.185
τ00 (Level 2 variance)	0.310	0.298	0.287
ICC	0.609	0.608	0.608
Model Fit			
−2 Log Likelihood	2187.2	2170.5	2150.9
AIC	2213.2	2200.5	2184.9
BIC	2253.9	2254.2	2251.6
Marginal R^2^	0.296	0.313	0.332
Conditional R^2^	0.661	0.673	0.686

Standard errors in parentheses. All continuous predictors are grand-mean centered

Level 1 *N* = 1,110 observations (185 participants × 6 time points)

Level 2 *N* = 185 participants

*p < .05, **p < .01, ***p < .001

## Discussion

### Interpretation of findings

The present study offers a nuanced examination of the intricate relationships between metacognitive processes, situational factors, and individual differences in the context of clinical decision-making among nursing students. The findings provide a multifaceted understanding of how these elements interact and evolve throughout nursing education.

The results demonstrate that each phase of metacognition contributes uniquely to clinical decision-making, with metacognitive regulation emerging as the strongest predictor (β = 0.188, *p* < 0.001 for quality; β = 0.168, *p* < 0.001 for efficiency). This hierarchical pattern strongly supports Nelson’s metacognitive model [47], which theorizes that monitoring and control processes interact to guide cognitive performance. Our findings extend Nelson’s model by quantifying the relative contributions of each metacognitive component in clinical settings: regulation showed the strongest effect (β = 0.188), followed by monitoring (β = 0.165) and evaluation (β = 0.141). This pattern contrasts with Schraw and Dennison’s [28] conceptualization of equal metacognitive components, suggesting domain-specificity in metacognitive functioning. Specifically, in nursing contexts, our data shows that regulatory processes account for 18.8% of the variance in decision quality, compared to 16.5% for monitoring and 14.1% for evaluation, highlighting the paramount importance of adaptive control processes in clinical decision-making.

The longitudinal analysis revealed distinct developmental trajectories, with decision-making efficiency showing a steeper growth rate (mean slope = 0.081, *p* < 0.001) compared to quality (mean slope = 0.065, *p* < 0.001). This finding both supports and extends Benner’s [48] novice-to-expert framework. While Benner proposed a general progression in nursing expertise, our results provide empirical evidence for differential rates of development across specific cognitive skills. The faster development of efficiency (slope difference = 0.016, *p* < 0.05) posits that procedural fluency develops before deep conceptual understanding. Our data specifically shows that efficiency gains were most pronounced in the first three months (β = 0.092, *p* < 0.001) before plateauing, while quality improvements showed a more gradual, sustained trajectory.

Our analysis of situational factors revealed that task complexity had a stronger negative impact on decision-making quality (β = −0.129, *p* < 0.001) than efficiency (β = −0.113, *p* < 0.001), while time pressure more strongly affected efficiency (β = −0.121, *p* < 0.001) than quality (β = −0.103, *p* < 0.001). These findings provide novel empirical support for Kahneman’s [49] dual-process theory in clinical contexts. The differential effects align with specific predictions of dual-process theory: System 2 processing, necessary for maintaining decision quality, showed greater susceptibility to complexity (evidenced by the larger negative coefficient), while time pressure pushed decision-making toward System 1 processing (shown by the stronger effect on efficiency). This extends previous work by quantifying the relative impact of different situational constraints on specific aspects of clinical decision-making.

The moderating effects of metacognitive processes on situational factors (regulation × task complexity: β = 0.068, *p* < 0.01; monitoring × time pressure: β = 0.052, *p* < 0.05) provide empirical support for Hammond’s Cognitive Continuum Theory [50]. While Hammond proposed that individuals adjust their cognitive mode based on task characteristics, our results specify the mechanisms of this adjustment. The stronger moderating effect of regulation compared to monitoring suggests that the ability to control cognitive processes plays a more crucial role in adaptation than awareness alone. This finding advances cognitive continuum theory by identifying specific metacognitive processes that facilitate cognitive flexibility.

Individual differences in cognitive style showed significant moderation effects on the relationship between metacognitive processes and decision-making outcomes (cognitive style × regulation: β = 0.055, *p* < 0.01; cognitive style × monitoring: β = 0.047, *p* < 0.05). These interactions provide specific support for Epstein’s [51] cognitive-experiential self-theory by demonstrating how individual processing preferences moderate the effectiveness of different metacognitive strategies. Notably, analytical cognitive style showed stronger positive interactions with regulation (β = 0.055) compared to monitoring (β = 0.047), suggesting that systematic thinkers benefit more from metacognitive control strategies.

The role of emotional intelligence emerged as a significant moderator, with stronger effects on regulation (β = 0.048, *p* < 0.05) than monitoring (β = 0.045, *p* < 0.05). These findings extend Damasio’s somatic marker hypothesis [52] by specifying how emotional intelligence enhances metacognitive processes. Our data indicates that emotional intelligence particularly strengthens the relationship between regulatory processes and decision outcomes (interaction effect: β = 0.048, *p* < 0.05), suggesting that emotional awareness facilitates more effective cognitive control. This provides the first quantitative evidence linking emotional intelligence to specific metacognitive processes in clinical decision-making.

Integrating these findings offers a deeper understanding of clinical decision-making in nursing by highlighting the interplay between metacognitive processes, situational factors, and individual characteristics. This perspective emphasizes that effective clinical decision-making may emerge from the adaptive application of metacognitive skills, particularly regulation, in response to varying situational demands and individual cognitive and emotional tendencies.

### Implications for nursing education

The findings of this study offer several important implications for nursing education, particularly in the development of metacognitive skills and clinical decision-making abilities. These implications extend beyond traditional pedagogical approaches and suggest a need for more nuanced, targeted interventions that consider the multifaceted nature of clinical reasoning.

Enhancing metacognitive skills in nursing curricula requires a differentiated approach that addresses each phase of metacognition. For monitoring skills, educational strategies could include structured reflection exercises and the use of think-aloud protocols during simulated clinical scenarios. These techniques, as suggested by [53], can help students become more aware of their thought processes during clinical decision-making. Evaluation skills might be fostered through peer-review activities and case-based discussions that encourage critical analysis of decision-making processes. This aligns with the collaborative learning approaches advocated by [54] in her research on clinical judgment in nursing. The development of regulation skills, given their prominent role in clinical decision-making as revealed in this study, warrants particular attention. Implementing scaffolded problem-solving exercises that gradually increase in complexity could help students develop their ability to adjust cognitive strategies in real-time. This approach resonates with the concept of cognitive apprenticeship proposed by [55], which emphasizes the importance of guided practice in developing complex cognitive skills.

To improve the application of metacognitive skills under various situational conditions, nursing education programs could incorporate simulation scenarios that systematically manipulate task complexity and time pressure. This approach extends beyond traditional simulation practices by explicitly focusing on the metacognitive aspects of decision-making. For instance, debriefing sessions following simulations could include targeted discussions about how students monitored, evaluated, and regulated their thinking processes under different conditions. For complex scenarios, educational strategies might include concept mapping exercises that help students visualize and navigate intricate clinical situations. This technique, as demonstrated by [56], can enhance students’ ability to make connections between different pieces of information, a crucial aspect of decision-making in complex cases. Time-pressured scenarios could be addressed through rapid-response simulations followed by detailed metacognitive debriefings, allowing students to analyze and improve their decision-making processes under time constraints.

Incorporating considerations of individual differences into educational strategies represents a significant shift towards personalized learning in nursing education. Based on the study’s findings regarding cognitive styles, educators might offer a range of decision-making exercises that cater to both analytical and intuitive thinkers. For analytically inclined students, structured decision trees and algorithmic approaches might be emphasized, while intuitive thinkers might benefit more from pattern recognition exercises and case-based reasoning tasks. This differentiated approach aligns with the cognitive continuum theory proposed by [50] and could help students leverage their cognitive strengths while developing complementary skills. The role of emotional intelligence in metacognitive development and decision-making suggests the need for integrating emotional competence training into nursing curricula. This could involve exercises that enhance students’ ability to recognize and manage emotions in clinical contexts, as well as training in empathetic communication. Such an approach extends beyond traditional nursing education by explicitly linking emotional competence with clinical reasoning, as suggested by the work of [57] on the role of emotion in clinical decision-making.

Furthermore, the longitudinal findings of this study imply that metacognitive skill development should be viewed as a continuous process throughout nursing education. Rather than treating it as a discrete topic, metacognitive training could be integrated across the curriculum, with increasing complexity and autonomy as students’ progress. This progressive approach aligns with the novice-to-expert framework proposed by [48] and could help ensure that students’ metacognitive abilities develop in tandem with their clinical knowledge and skills.

## Conclusion

This study examined relationships between metacognitive processes, situational factors, and individual differences in clinical decision-making among third-year nursing students at a single institution in China. Our findings suggest distinct contributions of metacognitive phases—monitoring, evaluation, and regulation—to clinical decision-making quality and efficiency. Within our study context, metacognitive regulation emerged as a potentially important predictor of decision-making outcomes, while monitoring and evaluation showed smaller but significant effects. These findings contribute to our understanding of how different metacognitive processes might influence clinical reasoning development.

The analysis of situational factors suggested that task complexity and time pressure may affect decision-making in different ways. Task complexity showed a stronger negative association with decision-making quality, while time pressure appeared to have a greater impact on efficiency. These patterns align with existing theoretical frameworks about clinical reasoning, though further research would be needed to establish their generalizability across different educational and clinical contexts. Our findings also indicated that individual differences, particularly cognitive style and emotional intelligence, may moderate the relationship between metacognitive processes and decision-making outcomes. The longitudinal data suggested potential developmental patterns in how nursing students acquire and apply clinical reasoning skills, though these patterns would need validation in diverse educational settings.

Several important limitations should be considered when interpreting these results. Our reliance on self-report measures for metacognitive awareness and decision-making outcomes may have introduced response biases, including social desirability bias and the Dunning-Kruger effect. While we implemented multiple data collection methods and standardized scenarios, future research would benefit from incorporating more objective measures, such as think-aloud protocols during clinical decision-making, physiological measurements, independent expert evaluations of clinical performance, and direct observation of decision-making behaviors. Additionally, conducting this study in a single educational institution with a specific cultural context limits the generalizability of our findings.

The findings have implications for nursing education, suggesting potential benefits of incorporating metacognitive awareness into clinical training. Specific approaches might include structured reflection exercises during clinical simulations, targeted practice with varying levels of task complexity, individual coaching based on cognitive style preferences, and integration of emotional awareness training. However, the effectiveness of these educational approaches would need to be evaluated through rigorous intervention studies across diverse educational settings. This research contributes to the ongoing discussion about clinical decision-making in nursing education by examining specific relationships between metacognitive processes, situational factors, and individual differences. While our findings suggest potentially important patterns in how nursing students develop clinical reasoning skills, they should be viewed as preliminary evidence that requires further validation. Future research directions might include multi-site studies across different cultural and educational contexts, longitudinal studies extending beyond one academic year, intervention studies testing specific educational approaches, investigation of additional factors affecting clinical decision-making, and development and validation of more objective measurement tools.

## Data Availability

The datasets used and/or analyzed during the current study are available from the corresponding author on reasonable request.
